# Exploring the Diversity and Metabolic Potential of CO_2_ fixation Mediated by RubisCO in Prokaryotes in the Japan Collection of Microorganisms

**DOI:** 10.1264/jsme2.ME25035

**Published:** 2026-01-20

**Authors:** Arisa Nishihara, Shingo Kato, Moriya Ohkuma

**Affiliations:** 1 Japan Collection of Microorganisms (JCM), RIKEN BioResource Research Center, 3-1-1 Koyadai, Tsukuba, Ibaraki 305-0074, Japan

**Keywords:** CBB cycle, RubisCO, autotroph, metabolic prediction, photosynthesis

## Abstract

A genome anal­ysis is essential for identifying valuable microbial resources for future applications. In the present study, we exami­ned potential CO_2_-fixing microorganisms based on the presence of the Calvin–Benson–Bassham (CBB) cycle using 6,262 bacterial and 487 archaeal genomes from available cultures in the Japan Collection of Microorganisms (JCM), a well-established culture collection, in October 2023. A total of 306 strains (147 genera, eight phyla) carried CBB cycle genes, and a literature survey showed that 74 genera had experimental evidence of autotrophic growth while 73 lacked supporting information. A phylogenetic anal­ysis of the large subunit of ribulose-1,5-bisphosphate carboxylase/oxygenase (RbcL) identified diverse forms (IA, IB, IC, IE, I+α, II, and III) with distinct metabolic associations; IA was associated with sulfur species oxidation and formed IC with hydrogen oxidation. Genome-based metabolic predictions identified the potential for CO_2_ fixation in numerous species lacking experimental evidence. Our anal­yses indicate that members of *Actinomycetota* harboring IE RbcL were generally associated with hydrogen oxidation, possibly by using oxygen or nitrate as an electron acceptor. Additionally, 12 species in *Pseudomonadota* contained photosystem II reaction center proteins (PufL and PufM), suggesting phototrophic capabilities. However, the prediction of electron donors failed in some of these species. They may use the CBB cycle to regulate the intracellular redox balance under photoheterotrophic growth. The present results reveal unrecognized autotrophic potential in JCM strains and broaden our knowledge of the diversity of CO_2_-fixing microorganisms. Experimental validation will clarify their roles in the global carbon cycle and their potential for biotechnological applications towards environmental sustainability.

The Calvin–Benson–Bassham (CBB) cycle is one of the most fundamental biochemical pathways for CO_2_ fixation, accounting for ~95% of fixed carbon on Earth (Field *et al.*, 1998). The CBB cycle is catalyzed by a pivotal enzyme called ribulose-1,5-bisphosphate carboxylase/oxygenase (RubisCO) (Banda *et al.*, 2020; Schulz *et al.*, 2022), which has attracted attention for its potential in industrial gas fermentation and has been genetically engineered for the production of various compounds (*e.g.*, polyhydroxyalkanoates) in photosynthetic organisms (*e.g.*, microalgae) and chemolithoautotrophic bacteria (Pachauri and Mayer, 2015; Nangle *et al.*, 2020; Arora *et al.*, 2023). In addition to the CBB cycle (Benson *et al.*, 1948), six natural CO_2_ fixation pathways that convert atmospheric CO_2_ to organic carbon have been identified in autotrophic microorganisms: 1) the reverse tricarboxylic acid (rTCA) cycle including a variant (the reversed oxidative TCA cycle (roTCA)) (Evans *et al.*, 1966; Searcy and Lee, 1998); 2) the 3-hydroxypropionate (3HP) bi-cycle (Holo and Øvreås, 1987); 3) the 4-hydroxybutyrate/3-hydroxypropionate (4HB/3HP) cycle (Berg *et al.*, 2007); 4) the dicarboxylate/4-hydroxybutyrate (DC/4HB) cycle (Berg *et al.*, 2007; Huber *et al.*, 2008); 5) the reductive acetyl-CoA (Wood–Ljungdahl [WL]) pathway (Wood and Werkman, 1936); and 6) the reductive glycine pathway. Among these pathways (Sánchez-Andrea *et al.*, 2014), the CBB cycle is distributed in photosynthetic organisms not only in bacteria, but also in Eukaryotes, such as higher plants and eukaryotic alga, in addition to various chemolithoautotrophic bacteria.

The RubisCO family of proteins has been classified into multiple structural and functional forms, including forms I, II, III, and IV, which differ from each other in their catalytic efficiency, substrate specificity, and evolutionary history (Tabita *et al.*, 2008; Prywes *et al.*, 2023). Besides form IV RubisCOs, which are RubisCO-like proteins that catalyze the carboxylation of ribulose-1,5-bisphosphate, all forms of RubisCO are involved in CO_2_ fixation. Form II RubisCO is characterized by its poor affinity for CO_2_, indicating its adapted function in low-O_2_ and high-CO_2_ environments (Badger and Bek, 2008). The oxygenase activity of RubisCO competes with its carboxylase activity, which is fundamental for driving CO_2_ fixation. Accordingly, form I RubisCOs have recruited a small subunit, the RubisCO small subunit (RbcS), whose encoding gene (*rbcS*) typically colocalizes in an operon with the gene encoding the large subunit (*rbcL*). These evolutionary mechanisms are considered to have increased the specificity of RubisCO for CO_2_ in ancient eras before oxygen was abundant on Earth (Schulz *et al.*, 2022; Nishihara *et al.*, 2024).

Form I RubisCOs have been further classified into the following subgroups: IA and IC (mostly in *Pseudomonadota*), IB (in *Cyanobacteria*, green algae, and plants), ID (in eukaryotic algae), IE (in *Actinomycetota*), and the *Thermus* group (Schulz *et al.*, 2022). A recent phylogenetic anal­ysis of the *rbcL* gene revealed four early-diverging orthologs of form I RubisCO that lacked the *rbcS* gene: forms I’ (Banda *et al.*, 2020), I-Anaero (Schulz *et al.*, 2022), I-α, and I” (West-Roberts *et al.*, 2021). These variants, identified from metagenome-assembled genomes, may be the key to elucidating how the small subunit of form I RubisCOs evolved to increase CO_2_ affinity. Form III RubisCO has been associated with the pentose bisphosphate pathway in some methanogenic archaea lacking phosphoribulokinase (PRK) (Sato *et al.*, 2007; Aono *et al.*, 2012, 2015). However, it remains unclear whether these archaea perform autotrophy via this pathway because methanogens primarily rely on the Wood–Ljungdahl pathway for CO_2_ fixation (Kono *et al.*, 2017). More recently, form III RubisCO harboring PRK was reported in the chemolithoautotrophic bacterium, *Thermodesulfobium acidiphilum* (*Thermodesulfobiota*), suggesting the involvement of a modified CBB cycle in CO_2_ fixation (Frolov *et al.*, 2019). These findings indicate variations in the function of CBB-associated genes in microbial metabolism.

Among phototrophic bacteria, all purple sulfur bacteria, which belong to *Gammaproteobacteria*, have been reported to fix CO_2_ via the CBB cycle (Shimada and Takaichi, 2024). Among purple non-sulfur bacteria, which belong to *Alphaproteobacteria* and *Betaproteobacteria*, some species fix CO_2_ via the CBB cycle, but also grow well under heterotrophic conditions (Imhoff, 2009). RubisCO-mediated inorganic carbon assimilation in purple non-sulfur bacteria contributes to alleviating an over-reduced state (Shimada and Takaichi, 2024). Aerobic anoxygenic phototrophic bacteria (AAPB), which are widely distributed across *Alpha*-, *Beta*-, and *Gammaproteobacteria*, are obligate aerobes exhibiting heterotrophic growth; however, there is currently no evidence of autotrophic growth in these bacteria (Yurkov and Beatty, 1998). Light-stimulated CO_2_ uptake has been reported in some AAPB species, such as *Acidiphilium rubrum* (*Acetobacterales* and *Alphaproteobacteria*) (Kishimoto *et al.*, 1995) and *Erythrobacter longus* (*Sphingomonadales* and *Alphaproteobacteria*) (Shiba, 1984; Shiba and Harashima, 1986; Yurkov and Beatty, 1998). Although CO_2_ fixation through the CBB cycle is insufficient to sustain autotrophic growth in AAPB, it may supply additional organic carbon intermediates for heterotrophic growth (Yurkov and Beatty, 1998). Therefore, the presence of CBB-related functional genes does not always correspond to autotrophic growth in phototrophic microorganisms, particularly in AAPB. To comprehensively investigate the activity of the CBB cycle in these bacteria, AAPB needs to be thoroughly exami­ned under culture conditions, with a focus on the uptake of CO_2_ and/or CO_2_-independent growth.

Despite the increasing number of bacteria that harbor the potential for autotrophic growth being identified through genomic and/or metagenomic studies, few studies have focused on the relationship between large-scale genomic information and culture-based physiological characteristics (Garritano *et al.*, 2022). To effectively utilize microorganisms as biological resources (*e.g.*, for bio-manufacturing), it is essential to integrate genomic information with cultivation data. The Japan Collection of Microorganisms (JCM) is one of the most well-established culture repositories in the world. Similar to other culture collections, such as the Leibniz Institute DSMZ (https://www.dsmz.de) and the Korean Collection for Type Cultures (https://kctc.kribb.re.kr/en/), JCM maintains cultivable species for reproducible experiments, including well-characterized type and non-type strains. According to data from the World Data Centre for Microorganisms (accessed in July 2025), JCM has the second-largest culture collection of archaeal and bacterial type strains (8,599 type strains) after DSMZ (12,927 type strains). The International Code of Nomenclature of Prokaryotes (ICNP) states that the culture collection plays a defining role in the description of novel species, issuing certificates of the deposition and availability of microorganisms, which are required for publishing new taxa. In addition to maintaining (type) strains, culture collections, such as JCM, are responsible for sequencing the genomes of strains, contributing to data in the Type Strain Genome Database (https://gctype.wdcm.org), an initiative under the Global Catalogue of Microorganisms project. As of July 2025, this project has sequenced 6,141 genomes out of 27,107 available type species.

In the present study, we exami­ned (1) potential CO_2_-fixing microorganisms from genome-sequenced species in the JCM collection that harbor CBB cycle genes and (2) the forms of RubisCO in these organisms, integrating information from previously reported culture-based data where possible. Furthermore, based on genomic data, we predicted (3) the metabolic potential of species in which autotrophic growth has not yet been documented. The present results afford a foundation for the systematic integration of genomic and laboratory cultivation data, which will provide a robust framework for understanding the diversity and physiology of microorganisms. Moreover, this framework will enable the strategic utilization of microbial resources in basic research and biotechnological applications.

## Materials and Methods

### Annotation of genomes for identifying CBB cycle genes

The present study analyzed 6,749 available genomes among 13,937 strains held in the JCM collection (both type and non-type). Genomes were collected from the RefSeq database in the National Center for Biotechnology Information (NCBI) (https://www.ncbi.nlm.nih.gov; Table S1), which represents a broad and taxonomically diverse set of microorganisms. For example, when referenced against the Genome Taxonomy Database Release 226 (April 2025), these genomes in the dataset covered 37 of the 51 valid phyla (1,616 of the 3,558 genera). Only 14 validated phyla were‍ ‍not covered, including *Abditibacteriota*, *Atribacterota*, *Caldisericota*, *Calditrichota*, *Chlamydiota*, *Chlorobiota*, *Chrysiogenota*, *Coprothermobacterota*, *Cyanobacteriota*, *Dictyoglomota*, *Fibrobacterota*, *Thermomicrobiota*, and *Vulcanimicrobiota* (taxonomy following NCBI).

To evaluate the genetic capacity for the CBB cycle, the collected genomes were annotated using METABOLIC (v4.0) software against the implemented hidden Markov model databases (the Kyoto Encyclopedia of Genes and Genomes [KEGG], KOfam, Pfam, TIGRfam, and custom hidden Markov models) with default options (Zhou *et al.*, 2022). According to the annotation results, species were deemed to potentially harbor CBB cycle genes if they possessed all four selected KEGG module step hits: M00165+01 (K00855 [PRK, *prkB*]), M00165+02 (K01601 [*rbcL*, *cbbL*]), M00165+03 (K00927 [PGK]), and M00165+04 (K05298 [GAPA], K00150 [*gap2*, *gapB*], or K00134 [GAPDH, *gapA*]).

### Prediction of the metabolic potential of putative autotrophs

Regarding species predicted to harbor CBB cycle genes through a genomic annotation anal­ysis, their cultivation conditions for CO_2_ fixation (electron donors and electron acceptors) were exami­ned by conducting a literature survey of available publications on Google Scholar (https://scholar.google.co.jp), as of December 2024. One representative species of genera with evidence of autotrophic growth and all available strains of genera lacking evidence of autotrophic growth were selected. The metabolic potential for autotrophic growth of strains lacking evidence of autotrophic growth was predicted based on the presence of functional proteins, as annotated using METABOLIC software and cross-referenced using the MetaCyc database (Karp *et al.*, 2019; Table S2). Predictions focused on proteins associated with electron acceptors (oxygen availability or anaerobic respiration using sulfur species and nitrate) and electron donors (hydrogen and sulfur species; Table S2). The predicted protein-coding genes of RubisCO-harboring strains were annotated using the Prokka tool (v1.14; Seemann, 2014). Additionally, photosynthesis-related genes encoding type-II reaction center proteins (*pufM* and *pufL*) were identified through a BLAST homology search against biochemically characterized proteins registered in the SwissProtKB database (Boutet *et al.*, 2007).

### Phylogenetic anal­ysis of the *rbcL* gene

The sequences of the *rbcL* gene were collected from the set of predicted protein-coding genes from species in the JCM collection. To construct a phylogenetic tree, representative sequences were selected through clustering at an 80% identity threshold using the CD-HIT tool (v.4.8.1; Fu *et al.*, 2012). Clustering was performed among representative sequences of the same genus lacking evidence of autotrophic growth, as confirmed through a literature survey. The selected sequences were aligned using the MAFFT program (v7.427) with the options “--localpair” and “--maxiterate 1000” (Katoh *et al.*, 2005), and the alignment was trimmed using the trimAl tool (v1.4.rev22; Capella-Gutiérrez *et al.*, 2009) with a gap threshold of 0.8. The final alignment was used as an input for maximum-likelihood phylogenetic estimations using the IQ-TREE software package (v2.1.3; Nguyen *et al.*, 2014). The tree was constructed using the profile mixture model, which was identified as the best-fit model by the ModelFinder tool (Kalyaanamoorthy *et al.*, 2017). The profile mixture model was implemented in IQ-TREE (v2.1.3), and 1,000 ultrafast bootstrap replicates were performed. Transfer bootstrap support values were calculated using the BOOSTER (v0.1.2) tool (Lartillot and Philippe, 2006).

## Results and Discussion

### CO_2_ fixation ability in species harboring CBB cycle genes

In the present study, we exami­ned the genomes of 6,262 bacterial and 487 archaeal strains, all of which are available in the JCM culture collection (Table S1). These strains were distributed across 1,731 genera within 37 phyla. A total of 306 strains (147 genera within eight phyla) were found to be potentially involved in the CBB cycle based on the presence of selected prodigal modules annotated using METABOLIC software (Zhou *et al.*, 2022) (Table S3). Most of these strains belonged to the phylum *Pseudomonadota* (207 species), with the second most represented phylum being *Actinomycetota* (82 species). Regarding archaea, only six species belonging to *Methanobacteriota* were identified.

We then conducted a literature survey to establish whether strains from the identified genera have been reported to fix CO_2_. The strains used for the literature survey are listed in Table S3 as representative species. Among the 147 genera, 74 contained strains with reported CO_2_ fixation capabilities, while the remaining 73 lacked supporting information. The genera that lacked supporting information encompassed 173 strains lacking evidence of autotrophic growth, including 15 strains reported to lack the ability for autotrophic growth. The strains in the JCM collection harboring CBB cycle genes and demonstrating CO_2_ fixation belonged to 54 genera (of 74 genera, 20 were omitted because the demonstrated species in these genera were unavailable in the JCM culture collection). In addition, possible electron donors and acceptors for CO_2_ fixation in these strains were exami­ned as described in Table S4 and summarized in Table 1 (discussed later). The genera reported to exhibit autotrophic growth were distributed across five phyla: *Actinomycetota*, *Thermodesulfobiota*, *Pseudomonadota*, *Thermodesulfobacteriota*, and *Methanobacteriota* (Table 1 and S4).

The results of the literature survey indicated that despite 21 genera within the phylum *Actinomycetota* being annotated as putative autotrophic bacteria based on genomic data, only three contained species reported to exhibit autotrophic growth: *Acidimicrobium* (*Acidimicrobiales*), *Mycolicibacterium* (*Mycobacteriales*), and *Pseudonocardia* (*Pseudonocardiales*) (Table 1, S3, and S4). All the species demonstrating autotrophic growth in these three genera were aerobic chemolithoautotrophs, utilizing hydrogen or ferrous irons as electron donors and oxygen as the electron acceptor (Table 1 and S4).

The phylum *Pseudomonadota* exhibited the most diverse range of metabolic types, including photosynthetic organisms (*e.g.*, purple sulfur or non-sulfur bacteria), reflecting the wide phylogenetic diversity within this phylum. Photoautotrophs were identified in the classes *Alphaproteobacteria* (*Rhodoplanes*, *Rhodopseudomonas*, and *Rhodobium* in *Hyphomicrobiales* and *Cereibacter*, and *Rhodobacter* and *Rhodovulum* in *Rhodobacterales*), *Betaproteobacteria* (*Rubrivivax* in *Burkholderiales*), and *Gammaproteobacteria* (*Caldichromatium*, *Halochromatium*, *Marichromatium*, *Thiohalocapsa*, and *Halorhodospira* in *Chromatiales*). These photoautotrophs reportedly utilize hydrogen or sulfur species (*e.g.*, thiosulfate and sulfide) as electron donors (Table 1 and S4). Among the chemolithoautotrophs identified, members of the classes “Zetaproteobacteria” (*Mariprofundus* and *Ghiorsea* in *Mariprofundales*) and *Hydrogenophilia* (*Hydrogenophilus* in *Hydrogenophilales*) required microaerophilic conditions (0.4–5% oxygen) for growth; members of the class *Alphaproteobacteria* (*Acetobacterales*, *Hyphomicrobiales*, *Rhodobacterales*, and *Rhodospirillales*) required microaerophilic conditions (1–10.5% oxygen) or aerobic conditions. Most bacterial species identified in the classes *Betaproteobacteria* (*Burkholderiales* and *Nitrosomonadales*) and *Gammaproteobacteria* (*Acidiferrobacterales*, *Chromatiales*, *Ectothiorhodospirales*, *Pseudomonadales*, and *Thiotrichales*) also required oxygen (ranging from microaerophilic to aerobic). *Betaproteobacteria* species specifically required microaerophilic conditions. Furthermore, some species—such as those in the genera *Noviherbaspirillum*, *Rubrivivax*, and *Thiomonas* (*Burkholderiales*)—exhibited chemolithoautotroph growth only under anaerobic conditions, utilizing nitrate as an electron acceptor (Table 1 and S4).

Species in the phyla *Thermodesulfobiota* (*Thermodesulfobium narugense*), *Methanobacteriota* (*Methanoculleus horonobensis* and *Methanospirillum hungatei*), and *Thermodesulfobacteriota* (*Dissulfurimicrobium hydrothermale*) have been reported to exhibit autotrophy under anaerobic conditions (Ferry *et al.*, 1974; Mori *et al.*, 2003; Shimizu *et al.*, 2013; Slobodkin *et al.*, 2016; Table‍ ‍1‍ ‍and S4). Since hydrogenotrophic methanogenic *Methanobacteriota* are known to fix inorganic carbon through methanogenesis via the Wood–Ljungdahl pathway (Borrel *et al.*, 2016), the CBB cycle is not their primary autotrophic mechanism. Regarding *D. hydrothermale*, a complete gene set for the Wood–Ljungdahl pathway was previously reported in the genome of this bacterial species (Yvenou *et al.*, 2022). While a previous study suggested that‍ ‍the CBB cycle gene set was incomplete in *D. hydrothermale*, our anal­ysis based on KEGG annotations (GhostKOALA) indicates that *D. hydrothermale* possesses a complete set of CBB cycle genes (Kanehisa *et al.*, 2016; see later discussion regarding form I-α RbcL).

### Forms and physiological characteristics of the RubisCO enzyme in autotrophy

**Fig. 1.**
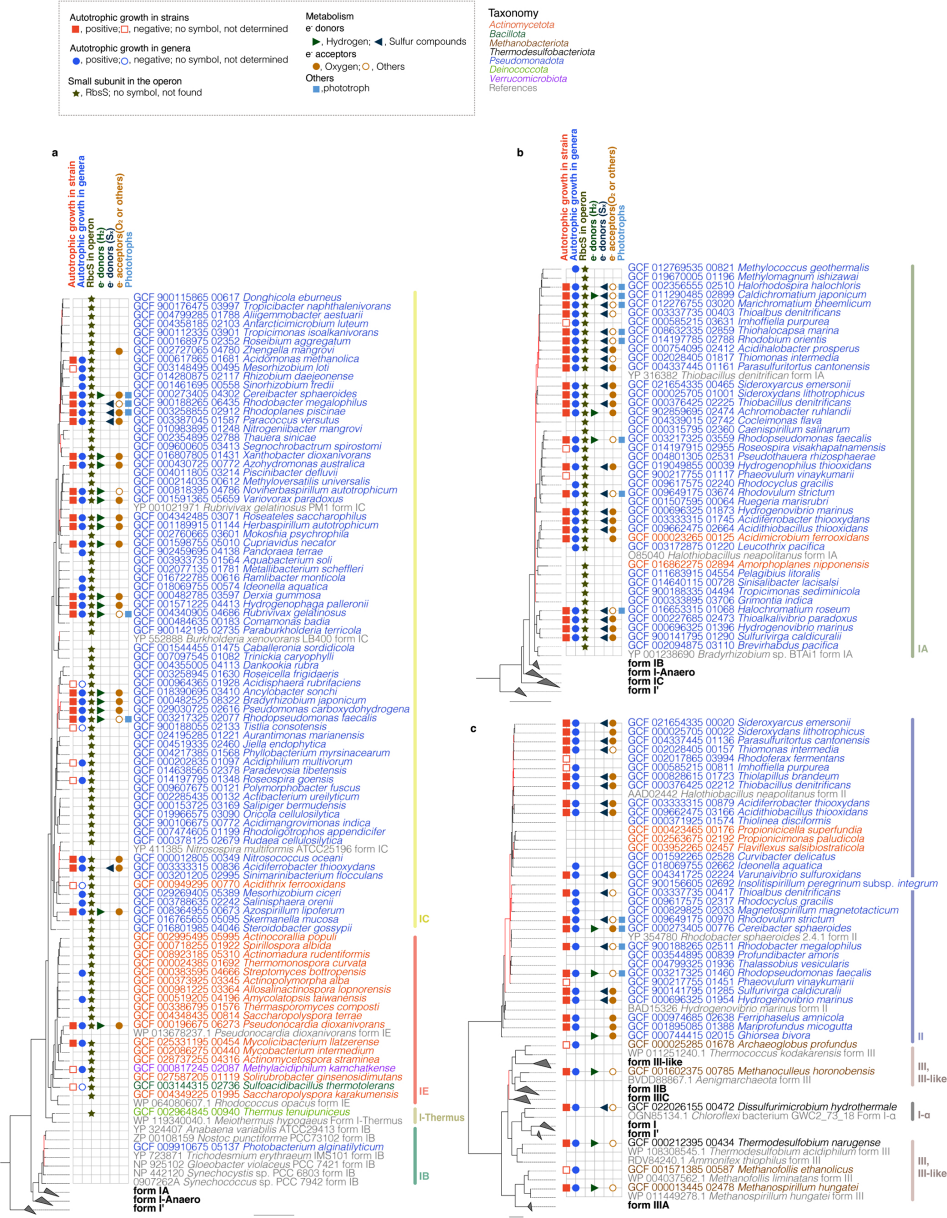
A maximum likelihood tree of amino acid sequences of RbcL. Species with confirmed CO_2_ fixation were included in the phylogenetic tree as representatives of their respective genera. The constructed phylogenetic trees of RbcL are shown without grouping branches of (a) form IC, IE, I-Thermus, IB, (b) form IA, (c) form II, III, III-like, and I+α. Reference sequences were collected from published data (Badger and Bek, 2008; Schulz *et al.*, 2022; Prywes *et al.*, 2023). The tree was constructed using IQ-TREE with the LG+C40+G4+F substitution model and 1,000 ultrafast bootstrap replicates. Input sequences were trimmed using trimAl (-gt 0.8), resulting in 211 sequences with 438 amino acid positions. Bootstrap branch support values were calculated using BOOSTER (v0.1.2) from ultrafast bootstrap (1,000 replicates). Unsupported branches with lower bootstrap values (≤90%) were collapsed, and branches with bootstrap values <95% were colored red. The scale bar indicates 1.0 changes per amino acid site. The presence of reported metabolic activity associated with autotrophic growth is shown for each strain, based on the data provided in Table S4. See Fig. S1 for the full tree.

To examine the forms of RubisCO found in strains in the JCM collection and their associated metabolic pathways, RbcL sequences were extracted and subjected to a phylogenetic clustering anal­ysis. The metabolic pathways related to‍ ‍autotrophic growth are shown in Fig. 1. The anal­ysis excluded form IV RubisCOs (RubisCO-like proteins) and archaeal PRK-lacking form III RubisCOs, which are associated with the pentose bisphosphate pathway (Sato *et al.*, 2007; Tabita *et al.*, 2008; Aono *et al.*, 2012, 2015). Various RubisCO forms were identified across multiple species in the JCM collection, including forms IA, IB, IC, IE, I+α, II, and III. In our constructed phylogeny, the reduced phylogeny (Fig. 1 and S1) and the phylogeny including all sequences (Fig. S2) both showed the same topology: the form I-Anaero clade branched off early from the form IA/IB groups and was positioned as a sister clade to the form IC/IE and *Thermus* clades. A recent study reported the positional instability of the form I-Anaero clade in phylogenetic reconstructions. However, our constructed tree exhibited the same topology as that of the tree constructed based on RbcS sequences (Schulz *et al.*, 2022; Liu *et al.*, 2023). Therefore, instead of discussing the tree topology, we focused on the phylogenetic distribution shown in Fig. 1.

Form IA RbcL was identified in 42 strains, of which 23 exhibited the ability to fix CO_2_ (Table S5, Fig. 1b). Form IB RbcL was previously reported in a *Cyanobacteria* species. In the present study, we detected form IB RbcL in *Photobacterium alginatilyticum* (*Pseudomonadota*), which suggests that this bacterium obtained *rbcL* via horizontal gene transfer from *Cyanobacteria* (Fig. 1a). Form IC was the most widely distributed, found in 97 strains, of which 22 exhibited the ability to fix CO_2_ (Table S5, Fig. 1a). Form IE, associated with the *Actinomycetota* cluster, was identified in 61 strains, of which two exhibited the ability to fix CO_2_ (Table S5, Fig. 1a). Although *rbcL* and other CBB cycle genes were previously shown to be absent in some species (Müller *et al.*, 2016), all eight species in the JCM collection of the genus *Thermus* possessed the *rbcL* gene belonging to the form I *Thermus* clade, which is consistent with previous findings on other members of this genus (Müller *et al.*, 2016; Schulz *et al.*, 2022) (Fig. 1a). To the best of our knowledge, only heterotrophic growth has been observed in *Thermus* species lacking evidence of autotrophic growth despite the discovery of RubisCO-encoding genes in the genomes of these species. Form II was identified in 33 species, of which 19 have been reported to exhibit the ability to fix CO_2_ (Table S5, Fig. 1c). Form III RbcL was only detected in *T. narugense* (*Thermodesulfobiota*). The amino acid sequence of *T. narugense* RbcL shared 94.87% similarity with that of *T. acidiphilum* RbcL, which has been shown to fix CO_2_ through a RubisCO-mediated transaldolase variant of the CBB cycle (Fig. 1c). Since *T. narugense* possesses a complete gene set of CBB cycle genes, as described in the previous section, it may also be capable of fixing CO_2_ similar to *T. acidiphilum* (Frolov *et al.*, 2019).

Strains that possessed forms IA, IC, and II RbcL mainly belonged to *Pseudomonadota* (Fig. 1). Although most *Actinomycetota* RbcL belonged to form IE, the *rbcL* sequences from six *Actinomycetota* species belonged to form I (one in form IC, two in IA, and three in II). These sequences were grouped with those from *Pseudomonadota* in a later-diverging phylogenetic branch, which suggests that these members acquired *rbcL* via horizontal gene transfer from *Pseudomonadota*. Among the six *Actinomycetota* species, autotrophic growth has been tested in two species. Positive autotrophic growth was reported in *Acidimicrobium ferrooxidans* (form IA RbcL) (Clark and Norris, 1996); however, *Acidithrix ferrooxidans* (form IC RbcL) has been reported to lack the ability to fix CO_2_ (Fig. 1a; Jones and Johnson, 2015). In *Actinomycetota*, tested strains for autotrophic growth were limited in form I as well as in form IE.

A notable result of our phylogenetic anal­ysis is that *rbcL* from *D. hydrothermale* was classified under the form I-α clade. A BLAST search of its complete genome revealed that the bacterium did not possess the *rbcS* gene (GCF_022026155; Fig. 1c). Three orthologs of form I RbcL that do not coexist with RbcS have been identified from metagenome-assembled genomes, namely, I’(Banda *et al.*, 2020), I-Anaero (Schulz *et al.*, 2022), and I-α (West-Roberts *et al.*, 2021); however, these have not been identified in culturable species. A more detailed understanding of the mechanisms of action of these enzymes using culturable species will provide insights into the evolution of form I RbcL and the co-option of the small subunit. *P. alginatilyticum* (*Pseudomonadota*) also does not possess RbcS, and form IB *rbcL* was apparently acquired through horizontal gene transfer from *Cyanobacteria* (see above).

To investigate the relationships between metabolic diversity and RubisCO forms, we exami­ned metabolic information on autotrophic growth available in the literature, with a focus on electron donors (hydrogen or sulfur species) and acceptors as well as the capacity for photoautotrophy. Sixteen of the 22 species harboring form IC RbcL utilized hydrogen as an electron donor while four utilized sulfur species (*e.g.*, sulfur, thiosulfate, and S°) (Fig. 1a, Table S4). In contrast, of the 24 species carrying form IA RbcL, three utilized hydrogen and 20 utilized sulfur species as electron donors (Fig. 1c, Table S4). The remaining species utilized other electron donors, such as ferrous iron (Table S4). Additionally, although many species harboring form II RbcL do not carry form I RbcL, all of the reported photoautotrophs harboring form II RbcL also harbored form IA or IC. These results suggest that different RubisCO forms are associated with metabolic variations in the utilization of electron donors, such as hydrogen versus sulfur compounds, despite the two species belonging to different phylogenetic groups (*e.g.*, at the class and order levels).

To obtain a more detailed understanding of the ecological niches of RubisCO-harboring microbial strains, we exami­ned the relationship between RubisCO forms and the environments from which strains in the JCM collection were isolated, with a focus on strains carrying RbcL (Table S5). Although most of the strains carrying both forms IA and IC RbcL belonged the same phylum of *Pseudomonadota*, they differed in the distribution of the environments from which they were isolated. Of the 42 form IA–harboring strains, 18 were isolated from marine environments, 21 from terrestrial environments (including six from rhizospheres), two from engineered environments (*e.g.*, wastewater), and one from an organism-hosted environment. In contrast, of the 97 form IC–harboring strains, 16 were isolated from marine environments, 51 from terrestrial environments (including 46 from rhizospheres), 17 from organism-hosted environments, 11 from engineered environments, and one from food. Fourteen of the 17 form IC-harboring strains isolated from organism-hosted environments came from plant-associated environments (*e.g.*, roots). Collectively, strains isolated from plant-associated environments (roots, leaves, and the rhizosphere) accounted for 46 of the 97 form IC–harboring strains, in contrast to only six of the 42 form IA–harboring strains. These results suggest that form IC–harboring strains are more commonly associated with plant-related environments.

### Metabolic predictions of potential autotrophs

As described above, 173 of the 306 strains possessing RubisCO genes lacked the capacity for CO_2_ fixation or their CO_2_ fixation ability had not yet been experimentally tested. Moreover, 143 of the 173 species belonged to genera in which no autotrophic strains have been reported (Table S5). Of the 143 strains, 77 encompassing 55 genera belonged to the phylum *Pseudomonadota*, 58 encompassing 16 genera to the phylum *Actinomycetota*, and seven from one genus to the phylum *Deinococcota*. We exami­ned the metabolic potential of these species to predict autotrophic conditions, with a focus on electron acceptors (oxygen availability or anaerobic respiration using sulfur species and nitrate) and donors (hydrogen and sulfur species; Table S5).

To identify species that utilized hydrogen as an electron donor, we exami­ned their genomes for the presence of genes encoding uptake hydrogenases. Based on the annotation results obtained using the METABOLIC software tool, hydrogenases annotated as Groups A2 and A3 ([FeFe]-hydrogenases) and Groups 1, 2a, 2b, 2c, 2d, 2e, 4h, and 4i ([NiFe]-hydrogenases) were selected as uptake hydrogenases (Søndergaard *et al.*, 2016; Zhou *et al.*, 2022). [NiFe]-hydrogenase genes were detected in 120 species, whereas [FeFe]-hydrogenase genes were only detected in one species. Nearly half of the identified species possessed the [NiFe]-hydrogenase gene, which indicates their potential utilization of hydrogen as an electron donor (Table 2 and S5). Among the 54 form IE-harboring strains, 33 contained the [NiFe]-hydrogenase gene, whereas only four contained genes involved in using sulfur species as an electron donor. Regarding form IA, previous studies showed a predominance of bacteria utilizing sulfur species as an electron donor (described above, also shown in Fig. 1b). However, among the strains belonging to genera in which CO_2_ fixation has not yet been demonstrated, the number of strains potentially utilizing sulfur species versus hydrogen as an electron donor was similar—nine species harbored sulfur species oxidation genes and seven harbored hydrogen oxidation genes (Table S5). Most of the analyzed strains belonging to the phylum *Pseudomonadota* were found to possess key genes encoding both microaerophilic-type terminal oxidases, which have high affinity for oxygen (heme–copper oxidases [HCOs] family C types [*ccoON*] and cytochrome *bd*-type oxidases [*cydAB*]), and aerobic-type terminal oxidases, which have low affinity for oxygen (HCOs-A type terminal oxidase [*coxAB* or *cyoABCD*]) (Morris and Schmidt, 2013). In contrast, species from other phyla, particularly *Bacillota* and *Deinococcota*, generally possessed either one of these terminal oxidase types, but not both (Table 2).

In the phylum *Pseudomonadota*, the highest number of species was found in the genus *Paraburkholderia* (*Burkholderiales*) at nine species, followed by the genus *Jiella* (*Hyphomicrobiales*) at four species (Table S5). The remaining genera contained three or fewer species. In this phylum, hydrogen and sulfur species appeared to mainly be used as electron donors and oxygen as an electron acceptor under chemolithoautotrophic conditions. A diverse range of species have the potential to fix CO_2_ through the CBB cycle, including 11 species that belong to monotypic genera under the classes *Alphaproteobacteria* and *Gammaproteobacteria*. Species from the class *Alphaproteobacteria* were *Tistlia* (form IA), *Zhengella* (form IC), *Segnochrobactrum* (form IC), *Dankookia* (form IC), *Profundibacter* (form II), *Thalassobius* (form II), and *Insolitispirillum* (form II). Species from the class *Gammaproteobacteria* were *Methylomagnum* (form IA), *Cocleimonas* (form IA), *Metallibacterium* (form IC), and *Rudaea* (form IC) (Table S5).

In the phylum *Actinomycetota*, all species possessed IE RbcL, except those in the genera *Flaviflexus* (*Actinomycetales*), *Amorphoplanes* (*Micromonosporales*), *Propionicicella*, and *Propionicimonas* (*Propionibacteriales*), which possessed IA or II RbcL. In this phylum, the genus containing the largest number of potential autotrophic species was *Actinomadura* (*Streptosporangiales*) with 24 species, followed by *Mycobacterium* (*Mycobacteriales*) with seven and *Actinomycetospora* (*Pseudonocardiales*) and *Thermomonospora* (*Streptosporangiales*) with five each. All *Actinomadura* species possessed the gene for low-affinity terminal oxidase (HCOs-A [*coxA*/*coxB*]), and 18 species additionally carried the genes encoding cytochrome *bd*-type oxidases (*cydA*/*cydB*), which are high-affinity terminal oxidases, suggesting the ability to grow under microaerophilic to aerobic conditions. The sulfide oxidation gene (*sqr*) was found in two species that possessed cytochrome *bd*-type oxidases (*cydA*/*cydB*). These species may grow autotrophically with sulfur species as an electron donor under microaerophilic to aerophilic conditions. Of the 15 species that carried the [NiFe]-hydrogenase gene, 12 also harbored nitrate reduction genes (*narG*/*narH*), suggesting CO_2_ fixation via hydrogen oxidization using hydrogen as an electron donor and oxygen as an electron acceptor under aerobic conditions or via nitrate reduction using hydrogen as an electron donor and nitrate as an electron acceptor. The same metabolic pathways are considered to be involved in *Actinomycetospora* and *Thermomonospora* species because all the species in these two genera possessed genes for high-affinity terminal oxidases and almost half possessed the genes for [NiFe]-hydrogenase and nitrate reduction (*narG*/*narH*).

The genus *Thermus* consists of thermophilic species that have been isolated worldwide in geothermal environments (≥55°C) with a pH range of 5.0 to 10.5. These species have demonstrated heterotrophic growth, but some have been reported to harbor complete sets of CBB cycle genes (Da Costa *et al.*, 2006; Müller *et al.*, 2016). All RbcL sequences from *Thermus* were grouped in the same cluster, as shown in a previous study (Müller *et al.*, 2016; Schulz *et al.*, 2022), and the genome anal­ysis revealed that species in this genus carried the gene for low-affinity terminal oxidases (*coxA*/*coxB*). Some species of the genus had genes for complete thiosulfate oxidation (*soxABXYZ*), whereas others had an incomplete set of genes lacking only *soxB*. Chemolithoautotrophic growth was previously reported in *Thermus sediminis* under conditions involving the oxidation of thiosulfate to sulfate; however, this characteristic was only described in the abstract and, thus, limited information was provided (Zhou *et al.*, 2018). Our genome anal­ysis also suggests that if CO_2_ fixation occurs, it is expected to take place under thiosulfate oxidation conditions.

Regarding the autotrophic potential of phototrophic bacteria, since the presence of CBB cycle genes does not always correspond to active CO_2_ fixation in phototrophs (Shimada and Takaichi, 2024), we also exami­ned photosynthesis-related genes to obtain for further insights into genera that have not been demonstrated to exhibit autotrophic growth at the genus level. Through a homologous BLAST search, we identified the *pufL* and *pufM* genes, which encode a type II reaction center involved in anoxygenic photosynthesis, in 12 species belonging to the phylum *Pseudomonadota*. These species belonged to eight genera, seven from the class *Alphaproteobacteria* (*Jiella*, *Aliigemmobacter*, *Phaeovulum*, *Dankookia*, *Skermanella*, *Polymorphobacter*, and *Rhodoligotrophos*) and one from the class *Betaproteobacteria* (*Piscinibacter*). All these species carried form IC RubisCO, except *Phaeovulum vinaykumarii*, which carried genes for both form IA and II RubisCOs. However, *P. vinaykumarii* has been reported to lack the ability to grow autotrophically under photosynthetic conditions (Srinivas *et al.*, 2007). Some autotrophic growth has‍ ‍been reported in *Rhodobacter* and *Rhodovulum* (*Paracoccaceae*, *Rhodobacterales*) species.

In the genus *Jiella* (*Alphaproteobacteria*), four species were identified as potential phototrophs, representing the highest number of species found among the genera analyzed. Genes involved in hydrogen or sulfur species (such as sulfide and thiosulfate) were not detected in the genomes of these species (Table S5), requiring further anal­yses to predict the metabolism of autotrophic growth. Alternatively, it is possible that *Jiella* utilizes the CBB cycle under heterotrophic growth conditions and does not involve autotrophic growth, as reported in AAPB (Yurkov and Beatty, 1998). In‍ ‍*Alphaproteobacteria*, *Hyphomicrobiales* containing *Jiella*‍ ‍(*Aurantimonadaceae*) and *Rhodoligotrophos* (*Parvibaculaceae*) accommodate multiple photoautotrophic purple non-sulfur bacteria (*e.g.*, *Rhodobium* and *Rhodopseudomonas*) (Table S4) and AAPB (*e.g.*, *Methylorubrum*) (Shimada and Takaichi, 2024). In *Acetobacterales*, which includes *Dankookia*, lacking the genes involved in electron donor utilization by *Jiella* (Table S5), studies have reported AAPB (*e.g.*, *Acidisphaera* and *Craurococcus*) and purple non-sulfur bacteria (*e.g.*, *Rhodopila* and *Rhodovastum*) (Shimada and Takaichi, 2024).

## Conclusion

The present study identified previously unrecognized autotrophic potential by predicting the CBB cycle in diverse accessible species in the JCM collection through an integrated anal­ysis of genome data and literature surveys. We clarified which CO_2_ fixation capabilities have been experimentally demonstrated and what remains unclear regarding the CO_2_ fixation potential of the isolated species. These results provide a foundation for future research focused on confirming the autotrophic capability of these species through further experimental anal­yses.

## Citation

Nishihara, A., Kato, S., and Ohkuma, M. (2026) Exploring the Diversity and Metabolic Potential of CO_2_ fixation Mediated by RubisCO in Prokaryotes in the Japan Collection of Microorganisms. *Microbes Environ ***41**: ME25035.

https://doi.org/10.1264/jsme2.ME25035

## Supplementary Material

Supplementary Material 1

Supplementary Material 2

Supplementary Material 3

## Figures and Tables

**Table 1.** Summary of autotrophs and representative growth conditions for each genus in the JCM culture collection.

Phylum	Order	Genus (representative JCM strain number)^¶^	Types*	Optimum oxygen concentration range**	optimum pH** (min, max)	optimum temp.** (min, max)	Electron donors in chemolithotrophs/photolithotrophs^†^	Electron acceptors in chemolithotrophs
*Actinomycetota*	*Acidimicrobiales*	*Acidimicrobium* (JCM 15462)	C	aerobic	2	45, 50	ferrous iron	oxygen
	*Mycobacteriales*	*Mycolicibacterium* (JCM 16229)^#1^	C	ND	ND	22, 30	hydrogen	oxygen
	*Pseudonocardiales*	*Pseudonocardia* (JCM 13855)	C	aerobic	7	30	hydrogen	oxygen
*Pseudomonadota*	*Acidiferrobacterales*	*Acidiferrobacter* (JCM 17358)	C	aerobic	1.2, 2	38	ferrous iron, pyrite, sulfur compounds	oxygen, iron (III)
	*Acidithiobacillales*	*Acidithiobacillus* (JCM 3867)	C	aerobic	4.5	30	sulfur, thiosulfate	oxygen
	*Burkholderiales*	*Achromobacter *(JCM 20676), *Azohydromonas* (JCM 20723), *Cupriavidus* (JCM 11282)^#3^, *Derxia* (JCM 20996)^#4^, *Herbaspirillum* (JCM 21424), *Hydrogenophaga* (JCM 21412), *Noviherbaspirillum* (JCM 17723), *Roseateles* (JCM 15912), *Rubrivivax* (JCM 21318), *Thiomonas* (JCM 20425), *Variovorax* (JCM 20526)	C, P	anaerobic, aerobic, microaerophilic (2.8–12%)	5.5, 9.0	28, 37	hydrogen, sulfur, sulfur compounds	nitrate, oxygen
	*Chromatiales*	*Caldichromatium* (JCM 39101), *Halochromatium* (JCM 14151), *Halorhodospira* (JCM 39378), *Marichromatium* (JCM 13911), *Nitrosococcus* (JCM 30415), *Thioalkalivibrio* (JCM 11367), *Thiohalocapsa* (JCM 14780), *Thiolapillus* (JCM 15507)	C, P	anaerobic, microaerophilic (2%), aerobic	6, 10.5	20, 50	hydrogen, ammonium, sulfur, sulfur compounds	nitrate, oxygen
	*Ectothiorhodospirales*	*Acidihalobacter* (JCM 30709), *Thioalbus* (JCM 15568)	C	anaerobic, aerobic	2, 7.5	28, 37	sulfur, sulfur compounds, ferrous iron, sphalerite, chalcopyrite, arsenopyrite, galena	nitrate, oxygen
	*Hydrogenophilales*	*Hydrogenophilus* (JCM 34254)	C	microaerophilic (5%)	7.0, 8.0	55	thiosulfate, sulfur	oxygen
	*Hyphomicrobiales*	*Ancylobacter* (JCM 32039), *Bradyrhizobium* (JCM 10834)^#3^, *Rhodobium* (JCM 9337), *Rhodoplanes* (JCM 14934), *Rhodopseudomonas* (JCM 11668), *Xanthobacter* (JCM 34666)	C, P	anaerobic, aerobic, microaerophilic (1%)	6.5, 8.5	22, 38	hydrogen, thiosulfate	oxygen
	*Mariprofundales*	*Mariprofundus* (JCM 30585), *Ghiorsea* (JCM 31637)	C	microaerophilic (0.4 or 1–3%)	6.0, 7.0	20, 25	ferrous iron, hydrogen	oxygen
	*Nitrosomonadales*	*Ferriphaselus* (JCM 18545), *Parasulfuritortus* (JCM 33645), *Sideroxyarcus* (JCM 39089), *Sideroxydans* (JCM 14762)^#5^, *Thiobacillus* (JCM 3870)	C	anaerobic, aerobic, microaerophilic (1–3%)	5.6, 7.5	25, 35	ferrous iron, sulfur compounds, sulfur	oxygen, nitrate, nitrite, nitrous oxide
	*Pseudomonadales*	*Pseudomonas* (JCM 12568)^#6^	C	microaerophilic (10%)	7	30	hydrogen	oxygen
	*Rhodobacterales*	*Cereibacter* (JCM 6121), *Paracoccus* (JCM 20754), *Rhodobacter* (JCM 14598), *Rhodovulum* (JCM 9220)	C, P	anaerobic, aerobic, microaerophilic (10.5%)	7-9.0	30, 37	hydrogen, sulfide, thiosulfate, sulfur	oxygen
	*Rhodospirillales*	*Azospirillum* (JCM 1247), *Varunaivibrio* (JCM 31027)	C	anaerobic, microaerophilic (2 or 5%)	5.5, 7.0	30, 37	hydrogen, sulfur, thiosulfate	nitrate, iron (III), oxygen
	*Acetobacterales*	*Acidomonas* (JCM 6891)^#2^	C	aerobic	3.0, 5.0	30, 32	methanol	oxygen
	*Thiotrichales*	*Hydrogenovibrio* (JCM 7688), *Sulfurivirga* (JCM 13439)	C	microaerophilic (1%,10%)	6, 6.5	37, 55	hydrogen, sulfur, thiosulfate, tetrathionate	oxygen
*Thermodesulfobacteriota*		*Dissulfurimicrobium* (JCM 19990)	C	anaerobic	6.0, 6.2	50, 52	sulfur (disproportionation)	sulfur
*Thermodesulfobiota*	*Thermoanaerobacterales*	*Thermodesulfobium* (JCM 11510)	C	anaerobic	4.0, 6.5	50, 55	hydrogen	sulfate
*Methanobacteriota*	*Methanomicrobiales*	*Methanoculleus* (JCM 15517), *Methanospirillum* (JCM 10133)	C	anaerobic	6.6, 7.4	30, 42	hydrogen	carbon dioxide

^¶^, Representative species are indicated in Table S3. Full species names and corresponding data with references are provided in Table S4. *, Autotrophic growth under chemolithotrophic (C) or phototrophic (P) conditions. ** Data on optimum or tested oxygen concentrations under autotrophic conditions, temperature, or pH were extracted from the reference; ND indicates “Not determined” (could not be found in the literature survey). ^†^, Electron donors comprising various reduced sulfur compounds (*e.g.*, sulfide, thiosulfate, and tetrathionate) are collectively referred to as sulfur compounds. ^#1^ Autotrophic growth was not observed under conditions with CO_2_ and O_2_ in the gas phase. ^#2^ Growth initially depended on autotrophic CO_2_ fixation, and CO_2_ assimilation was subsequently coupled with the ribulose monophosphate (RuMP) pathway during the early to mid-log phases of methylotrophic growth. ^#3^ The strain used in the experiment demonstrating autotrophic growth is different from the JCM strain. ^#4^ Source information is not available. ^#5^ Unvalidated species. ^#6^ Autotrophic growth via CO_2_ oxidation has been reported instead of CO_2_ fixation.

**Table 2.** Summary of the strain number corresponding to the predicted metabolism based on genome information. The species used in this anal­ysis were derived from genera lacking evidence of autotrophic growth. Light gray highlights indicate the total number for each phylum. Details of the genes used for metabolic predictions are given in Table S1. The full strain names and corresponding data are provided in Table S5.

Taxonomy	Total number of strains analyzed	RubisCO forms							Electron donors			Electron acceptors				Photosynthesis (pufL/pufM)
		IA	IB	IC	IE	I_thermus	II		H2 oxidation	Sulfur species oxidation		Microaerophilic (HCOs-B,C)	Aerobic (HCOs-A)	Sulfur species reduction	Nitrate reduction	
total counts of strains	144	14	1	58	54	7	10		63	46		123	131	32	77	12
*Actinomycetota* (Phylum)	58	1		1	53		3		33	3		51	49	2	34	
*Acidimicrobiales*	1			1									1	1		
*Actinomycetales*	1						1					1	1	1	1	
*Micromonosporales*	1	1										1			1	
*Mycobacteriales*	7				7				7	1		7			4	
*Propionibacteriales*	7				5		2					7	6		1	
*Pseudonocardiales*	7				7				3			7	7		4	
*Solirubrobacterales*	2				2				2			2	2		1	
*Streptosporangiales*	32				32				21	2		26	32		22	
*Bacillota* (Phylum)	1				1					1		1		1		
*Alicyclobacillales*	1				1					1		1		1		
*Deinococcota* (Phylum)	7					7			1	4			7	7	4	
*Thermales*	7					7			1	4			7	7	4	
*Pseudomonadota* (Phylum)	78	13	1	57			7		29	38		71	75	22	39	12
*Acetobacterales*	3			3						1		3	3		1	1
*Burkholderiales*	20			18			2		4	6		17	18	1	8	1
*Hyphomicrobiales*	13			13					2	3		13	13	1	3	5
*Lysobacterales*	2			2					1	1		2	2			
*Methylococcales*	1	1								1		1	1			
*Nevskiales*	1			1					1			1	1	1		
*Nitrosomonadales*	2			2					2	1		2	2	2	2	
*Rhodobacterales*	16	5		9			3		8	15		14	15	15	11	2
*Rhodocyclales*	3	1		2					2	2		3	3		3	
*Rhodospirillales*	6	2		3			1		5	6		6	6		5	2
*Sphingomonadales*	1			1								1	1			1
*Steroidobacterales*	3			3					1			1	3		2	
*Thiotrichales*	2	1					1		2	2		2	2	2		
*Vibrionales*	5	3	1						1			5	5		4	
