# Supplementary figures and images for: Exploring the Diversity and Metabolic Potential of CO2 fixation Mediated by RubisCO in Prokaryotes in the Japan Collection of Microorganisms
Source: Microbes Environ. 2026 Jan 20;41(1):ME25035. doi: 10.1264/jsme2.ME25035 (PMC12999727; doi:10.1264/jsme2.ME25035)

Fig.S1  
Nishihara et al.

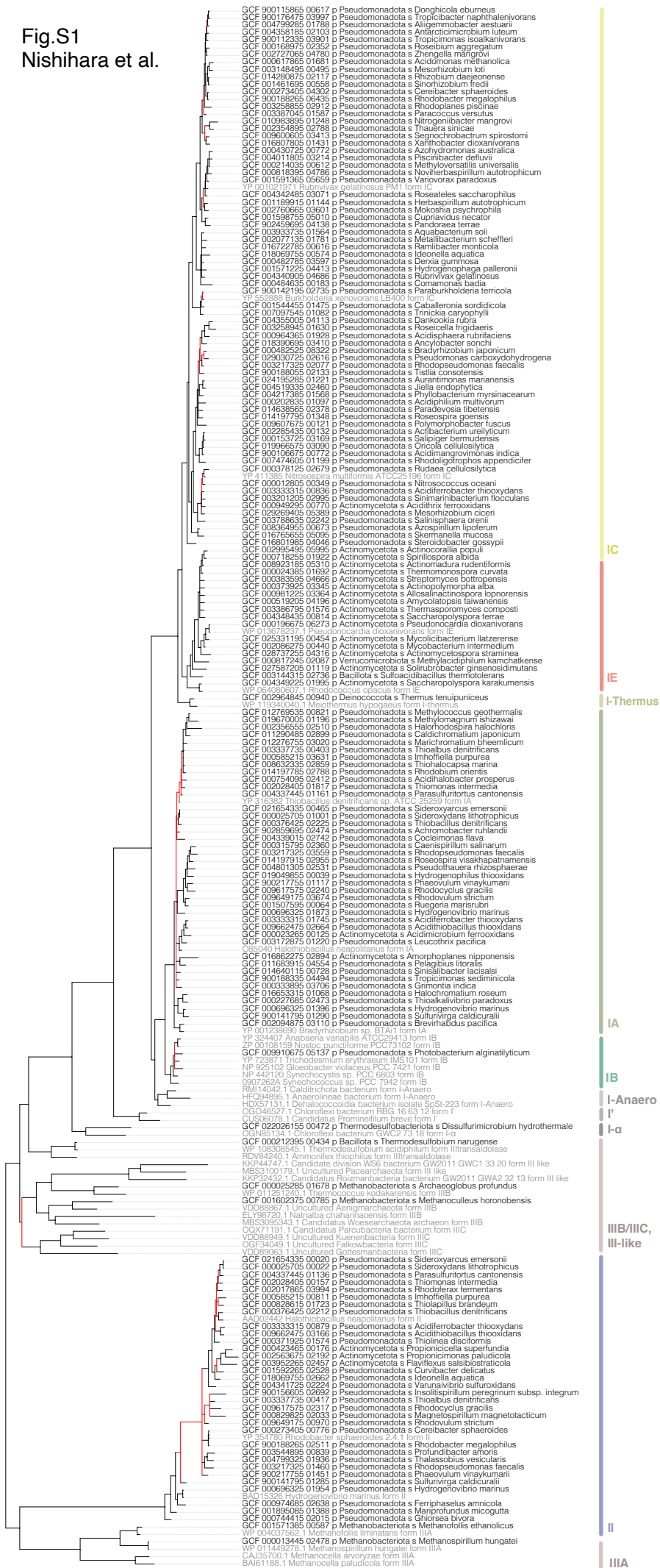

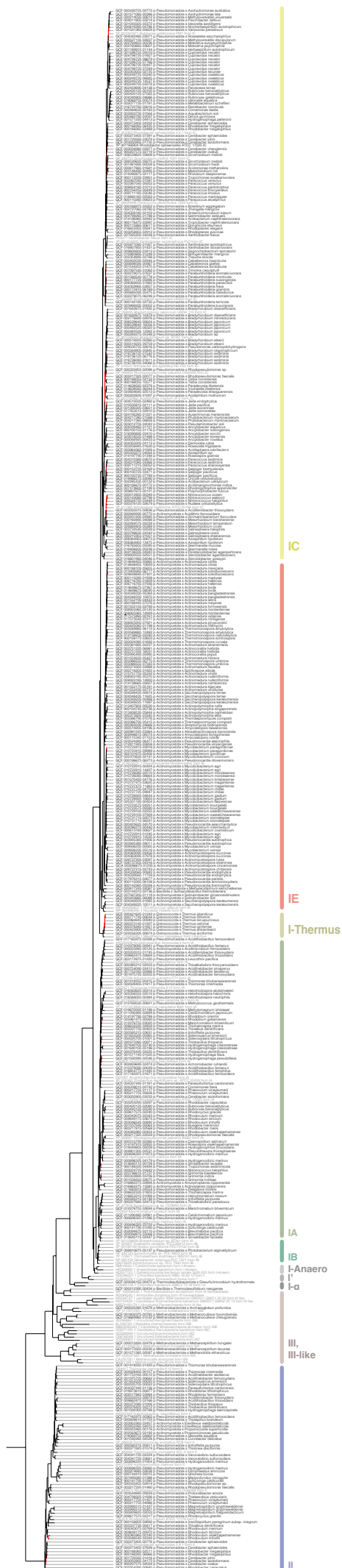

Supplement: Supplementary file 1 — Supplementary Material 1 [file 41_25035_s1.pdf]
