# Supplementary material for: Exploring the Diversity and Metabolic Potential of CO2 fixation Mediated by RubisCO in Prokaryotes in the Japan Collection of Microorganisms
Source: Microbes Environ. 2026 Jan 20;41(1):ME25035. doi: 10.1264/jsme2.ME25035 (PMC12999727; doi:10.1264/jsme2.ME25035)
Supplement: Supplementary file 3 — Supplementary Material 3 [file 41_25035_s3.docx]

**Supplemental table legends**

**Table S1.** Lists of species in JCM collection used for genome analysis

**Table S2**. Lists of key genes associated with electron acceptors and electron donors for metabolic prediction.

**Table S3.** List of microbial species analyzed in this study.

^*^, Species used for literature-based surveys, phylogenetic analysis, and metabolic potential analysis are marked with a "+".

^†^, For species whose CO_2_-fixation potential could not be determined from the literature data, "ND" (Not Determined) is indicated.

**Table S4**. Autotrophic species and representative growth conditions for each genus in the JCM culture collection. The summary is available in Table 1.

* Data on optimum temperature or pH were extracted from the reference; ND indicates "Not Determined" (could not be found in the literature survey).

**Table S5.** Summary of key gene sets for metabolic prediction, RubisCO forms in the genome, and isolation sources. Metabolic predictions were performed based on the criteria listed in Table S1a, b, c. The annotation of RubisCO forms is based on Fig.1, Fig. S1, and Fig. S2. A summary of the metabolic prediction counts for species with no reported evidence of autotrophic growth is provided in Table 2.

**Supplemental figure legends**

**Figure S1**. Maximum likelihood tree of the amino acid sequences of RbcL. With the exception of the grouping of RbcL forms, the tree is congruent with the one in Fig. 1.

**Figure S2.** Maximum likelihood tree of the amino acid sequences of RbcL. All the sequences from the strain used in this study were included for constructing the tree with reference sequences collected from published data (Badger and Bek, 2008; Prywes *et al*., 2023; Schulz *et al*., 2022). The tree was constructed using IQ-TREE with the LG+C60+G4+F substitution model and 1,000 ultrafast bootstrap replicates. Input sequences were trimmed using trimAl (-gt 0.8), resulting in 488 sequences with 466 amino acid positions. The bootstrap branch support values were calculated using BOOSTER (v0.1.2) from ultrafast bootstrap (1000 replicates). Unsupported branches with lower bootstrap values (≤90%) were collapsed, and branches with bootstrap values <95% were colored red. The scale bar indicates 1.0 changes per amino acid site.
